# 

**Published:** 1924-04

**Authors:** 


### CONTENTS.

| | Page |
|---|---|
| **The Evolutionary History of Renal Surgery and of Temporal Bone Surgery.** By J. LACY FIRTH, M.D., M.S. Lond., F.R.C.S. ... ... | 49 |
| **Cancer of the Breast.** By A. RENDLE SHORT, M.D., B.S., B.Sc., F.R.C.S. ... ... ... ... ... ... ... ... ... ... | 64 |
| **Grave Cardiac Symptoms due to Multiple Dental Abscess.** By W. R. ACKLAND, M.D.S. ... ... ... ... ... ... ... | 79 |
| **Reviews of Books** ... ... ... ... ... ... ... ... ... | 82 |
| **Editorial Notes** ... ... ... ... ... ... ... ... ... | 87 |
| **Meetings of Societies** ... ... ... ... ... ... ... ... | 88 |
| **Obituary:** R. G. POOLE LANSDOWN, M.D., B.S. ... ... ... .. | 89 |
| ALFRED JAMES HARRISON, M.B. Lond. ... ... ... ... | 93 |
| **Local Medical Notes** ... ... ... ... ... ... ... ... | 95 |

